# 2-Trifluoro­methyl-1*H*-benzimidazol-3-ium hydrogen sulfate

**DOI:** 10.1107/S1600536811048811

**Published:** 2011-11-30

**Authors:** Ming-Liang Liu

**Affiliations:** a College of Chemistryand Chemical Engineering, Southeast University, Nanjing 211189, People’s Republic of China

## Abstract

In the crystal of the title mol­ecular salt, C_8_H_6_F_3_N_2_^+^·HSO_4_^−^, cation-to-anion N—H⋯O hydrogen bonds generate [100] chains. Anion-to-anion O—H⋯O hydrogen bonds generate [001] helices and cross-link the chains into a three-dimensional network.

## Related literature

For a related structure and background to mol­ecular salts, see: Liu (2011 ▶).
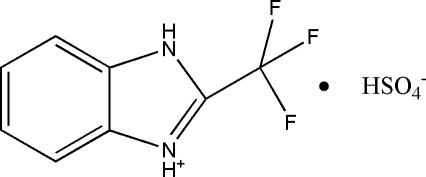


## Experimental

### 

#### Crystal data

C_8_H_6_F_3_N_2_^+^·HSO_4_^−^
*M* *_r_* = 284.22
Hexagonal, 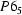

*a* = 9.4119 (13) Å
*c* = 21.960 (4) Å
*V* = 1684.7 (5) Å^3^
*Z* = 6
Mo *K*α radiation
μ = 0.34 mm^−1^
*T* = 293 K
0.20 × 0.20 × 0.20 mm

#### Data collection

Rigaku Mercury2 CCD diffractometer
Absorption correction: multi-scan (*CrystalClear*; Rigaku, 2005 ▶) *T*_min_ = 0.935, *T*_max_ = 0.935
14287 measured reflections
1977 independent reflections
1941 reflections with *I* > 2σ(*I*)
*R*_int_ = 0.038

#### Refinement

*R*[*F* ^2^ > 2σ(*F* ^2^)] = 0.052
*wR*(*F* ^2^) = 0.134
*S* = 1.11
1977 reflections
167 parameters
8 restraints
H atoms treated by a mixture of independent and constrained refinement
Δρ_max_ = 0.53 e Å^−3^
Δρ_min_ = −0.25 e Å^−3^
Absolute structure: Flack (1983 ▶), 957 Friedel pairs
Flack parameter: 0.03 (16)

### 

Data collection: *CrystalClear* (Rigaku, 2005 ▶); cell refinement: *CrystalClear*; data reduction: *CrystalClear*; program(s) used to solve structure: *SHELXS97* (Sheldrick, 2008 ▶); program(s) used to refine structure: *SHELXL97* (Sheldrick, 2008 ▶); molecular graphics: *SHELXTL* (Sheldrick, 2008 ▶); software used to prepare material for publication: *SHELXTL*.

## Supplementary Material

Crystal structure: contains datablock(s) I, global. DOI: 10.1107/S1600536811048811/hb6491sup1.cif

Structure factors: contains datablock(s) I. DOI: 10.1107/S1600536811048811/hb6491Isup2.hkl

Supplementary material file. DOI: 10.1107/S1600536811048811/hb6491Isup3.cml

Additional supplementary materials:  crystallographic information; 3D view; checkCIF report

## Figures and Tables

**Table 1:** Hydrogen-bond geometry (Å, °)

*D*—H⋯*A*	*D*—H	H⋯*A*	*D*⋯*A*	*D*—H⋯*A*
N2—H2*A*⋯O3^i^	0.86	1.85	2.707 (5)	173
N1—H1*A*⋯O4	0.86	1.88	2.740 (7)	174
O1—H1⋯O2^ii^	0.86 (2)	1.86 (6)	2.608 (7)	145 (9)

Symmetry codes: (i) 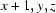; (ii) 
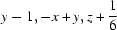
.

**Fig. 1.**
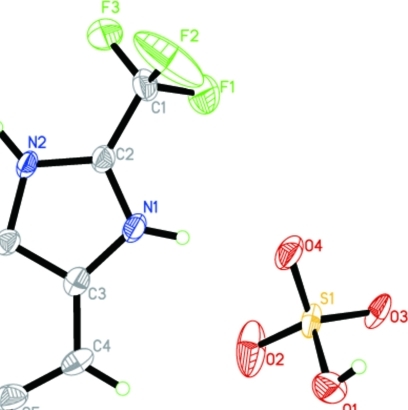
Fig. 1.The molecular structure of the title compound, showing 30% probability displacement ellipsoids.

**Fig. 2.**
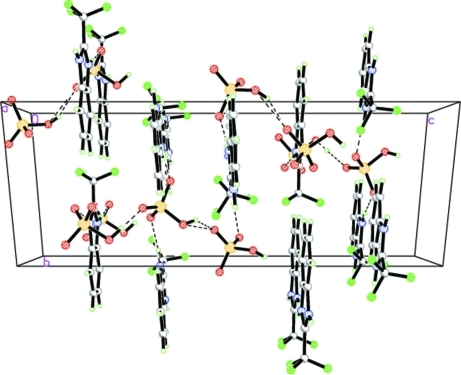
A view of the packing of the title compound, stacking along the a axis. Dashed lines indicate hydrogen bonds.

## supplementary crystallographic information

### Experimental

0.144 g(1 mmol) of 2-Trifluoromethl-1*H*-benzimidazole was firstly dissolved in 30 ml me thanol, to which 0.1 g (1 mmol) of sulfuric acid was then added to afford the solution without any precipitation under stirring at the ambient temperature. Colourless blocks of the title compound were obtained by the slow evaporation of the above solution after 2 days in air.

The dielectric constant of the compound as a function of temperature indicates that the permittivity is basically temperature-independent (*ε* = C/(T–T_0_)), suggesting that this compound is not ferroelectric or there may be no distinct phase transition occurring within the measured temperature within the measured temperature (below the melting point).

### Refinement

H atoms were placed in calculated positions (N—H = 0.89 Å; C—H = 0.93Å for C*sp^2^* atoms and C—H = 0.96Å and 0.97Å for C*sp^3^* atoms), assigned fixed *U*_iso_ values [*U*_iso_ = 1.2*U*eq(C*sp^2^*) and 1.5*U*eq(C*sp^3^*,*N*)] and allowed to ride.The H atom bonding with N was found with O—H bond distance of 0.8600Åin the difference electron density map.

### Crystal data

**Table:**

C_8_H_6_F_3_N_2_^+^·HSO_4_^−^	*F*(000) = 864
*M**_r_* = 284.22	*D*_x_ = 1.681 Mg m^−^^3^
Hexagonal, *P*6_5_	Mo *K*α radiation, λ = 0.71073 Å
Hall symbol: P 65	θ = 3.1–27.6°
*a* = 9.4119 (13) Å	µ = 0.34 mm^−^^1^
*c* = 21.960 (4) Å	*T* = 293 K
*V* = 1684.7 (5) Å^3^	Block, colorless
*Z* = 6	0.20 × 0.20 × 0.20 mm

### Data collection

**Table:**

Rigaku Mercury2 CCD diffractometer	1977 independent reflections
Radiation source: fine-focus sealed tube	1941 reflections with *I* > 2σ(*I*)
graphite	*R*_int_ = 0.038
CCD_Profile_fitting scans	θ_max_ = 25.0°, θ_min_ = 3.1°
Absorption correction: multi-scan (*CrystalClear*; Rigaku, 2005)	*h* = −11→11
*T*_min_ = 0.935, *T*_max_ = 0.935	*k* = −11→11
14287 measured reflections	*l* = −26→26

### Refinement

**Table:**

Refinement on *F*^2^	Secondary atom site location: difference Fourier map
Least-squares matrix: full	Hydrogen site location: inferred from neighbouring sites
*R*[*F*^2^ > 2σ(*F*^2^)] = 0.052	H atoms treated by a mixture of independent and constrained refinement
*wR*(*F*^2^) = 0.134	*w* = 1/[σ^2^(*F*_o_^2^) + (0.0593*P*)^2^ + 1.9666*P*] where *P* = (*F*_o_^2^ + 2*F*_c_^2^)/3
*S* = 1.11	(Δ/σ)_max_ < 0.001
1977 reflections	Δρ_max_ = 0.53 e Å^−^^3^
167 parameters	Δρ_min_ = −0.25 e Å^−^^3^
8 restraints	Absolute structure: Flack (1983), 957 Friedel pairs
Primary atom site location: structure-invariant direct methods	Flack parameter: 0.03 (16)

### Special details

**Table:**

Geometry. All e.s.d.'s (except the e.s.d. in the dihedral angle between two l.s. planes) are estimated using the full covariance matrix. The cell e.s.d.'s are taken into account individually in the estimation of e.s.d.'s in distances, angles and torsion angles; correlations between e.s.d.'s in cell parameters are only used when they are defined by crystal symmetry. An approximate (isotropic) treatment of cell e.s.d.'s is used for estimating e.s.d.'s involving l.s. planes.
Refinement. Refinement of *F*^2^ against ALL reflections. The weighted *R*-factor *wR* and goodness of fit *S* are based on *F*^2^, conventional *R*-factors *R* are based on *F*, with *F* set to zero for negative *F*^2^. The threshold expression of *F*^2^ > σ(*F*^2^) is used only for calculating *R*-factors(gt) *etc*. and is not relevant to the choice of reflections for refinement. *R*-factors based on *F*^2^ are statistically about twice as large as those based on *F*, and *R*- factors based on ALL data will be even larger.

### Fractional atomic coordinates and isotropic or equivalent isotropic displacement parameters (Å^2^)

**Table:**

	*x*	*y*	*z*	*U*_iso_*/*U*_eq_	
S1	0.09970 (14)	0.72553 (17)	0.16867 (6)	0.0442 (3)	
N1	0.4873 (5)	0.7391 (5)	0.1735 (2)	0.0418 (9)	
H1A	0.3843	0.7058	0.1770	0.050*	
N2	0.7169 (4)	0.7349 (5)	0.16388 (17)	0.0348 (8)	
H2A	0.7835	0.6981	0.1596	0.042*	
C3	0.6124 (6)	0.9017 (6)	0.1722 (2)	0.0418 (11)	
C8	0.7613 (5)	0.9011 (6)	0.1675 (2)	0.0371 (10)	
C4	0.6126 (7)	1.0512 (7)	0.1762 (3)	0.0508 (13)	
H4	0.5152	1.0535	0.1792	0.061*	
O3	−0.0637 (4)	0.6347 (5)	0.14218 (18)	0.0558 (11)	
F3	0.5450 (5)	0.4034 (5)	0.1391 (3)	0.0928 (15)	
C6	0.9082 (7)	1.1891 (7)	0.1712 (3)	0.0527 (13)	
H6	1.0066	1.2883	0.1710	0.063*	
O1	0.0866 (5)	0.8248 (5)	0.2230 (2)	0.0627 (11)	
C5	0.7592 (8)	1.1907 (7)	0.1755 (3)	0.0589 (15)	
H5	0.7623	1.2909	0.1780	0.071*	
F1	0.3262 (5)	0.4077 (6)	0.1346 (3)	0.116 (2)	
F2	0.4211 (10)	0.3991 (6)	0.2195 (2)	0.157 (3)	
O4	0.1589 (5)	0.6207 (5)	0.19150 (19)	0.0571 (11)	
C7	0.9120 (6)	1.0455 (7)	0.1670 (3)	0.0456 (11)	
H7	1.0103	1.0448	0.1641	0.055*	
C1	0.4609 (6)	0.4596 (7)	0.1670 (3)	0.0520 (13)	
O2	0.2184 (6)	0.8507 (9)	0.1300 (3)	0.110 (2)	
C2	0.5531 (5)	0.6434 (6)	0.1683 (2)	0.0373 (9)	
H1	0.041 (10)	0.754 (9)	0.251 (3)	0.10 (3)*	

### Atomic displacement parameters (Å^2^)

**Table:**

	*U*^11^	*U*^22^	*U*^33^	*U*^12^	*U*^13^	*U*^23^
S1	0.0252 (5)	0.0626 (9)	0.0458 (6)	0.0227 (5)	0.0080 (5)	0.0144 (6)
N1	0.0295 (19)	0.051 (2)	0.051 (2)	0.0252 (19)	−0.0012 (18)	−0.0049 (19)
N2	0.0243 (17)	0.044 (2)	0.040 (2)	0.0205 (16)	0.0009 (16)	−0.0062 (18)
C3	0.037 (2)	0.050 (3)	0.043 (3)	0.025 (2)	0.001 (2)	−0.002 (2)
C8	0.037 (2)	0.043 (3)	0.037 (2)	0.024 (2)	−0.006 (2)	−0.005 (2)
C4	0.057 (3)	0.058 (3)	0.059 (3)	0.044 (3)	−0.002 (3)	0.003 (3)
O3	0.0362 (19)	0.087 (3)	0.059 (2)	0.042 (2)	−0.0057 (16)	−0.015 (2)
F3	0.070 (2)	0.058 (2)	0.158 (4)	0.038 (2)	0.018 (3)	−0.012 (3)
C6	0.044 (3)	0.045 (3)	0.062 (3)	0.017 (2)	0.006 (3)	0.009 (3)
O1	0.056 (3)	0.048 (2)	0.079 (3)	0.022 (2)	−0.009 (2)	−0.012 (2)
C5	0.073 (4)	0.052 (3)	0.070 (4)	0.045 (3)	−0.005 (3)	0.005 (3)
F1	0.047 (2)	0.070 (3)	0.213 (6)	0.0161 (19)	−0.039 (3)	−0.044 (3)
F2	0.246 (6)	0.055 (3)	0.072 (3)	0.002 (3)	0.038 (4)	0.009 (2)
O4	0.0403 (19)	0.074 (3)	0.072 (3)	0.039 (2)	0.0007 (17)	0.007 (2)
C7	0.030 (2)	0.055 (3)	0.050 (3)	0.020 (2)	−0.002 (2)	0.003 (2)
C1	0.036 (3)	0.043 (3)	0.067 (3)	0.012 (2)	0.014 (3)	−0.001 (3)
O2	0.057 (3)	0.155 (5)	0.108 (4)	0.045 (3)	0.032 (3)	0.093 (4)
C2	0.030 (2)	0.040 (2)	0.042 (2)	0.0176 (19)	0.0001 (19)	−0.010 (2)

### Geometric parameters (Å, °)

**Table:**

S1—O2	1.429 (5)	C4—C5	1.348 (9)
S1—O4	1.444 (4)	C4—H4	0.9300
S1—O3	1.456 (4)	F3—C1	1.304 (6)
S1—O1	1.558 (5)	C6—C7	1.373 (8)
N1—C2	1.329 (6)	C6—C5	1.414 (8)
N1—C3	1.388 (6)	C6—H6	0.9300
N1—H1A	0.8600	O1—H1	0.86 (2)
N2—C2	1.341 (6)	C5—H5	0.9300
N2—C8	1.405 (6)	F1—C1	1.317 (8)
N2—H2A	0.8600	F2—C1	1.258 (8)
C3—C8	1.407 (6)	C7—H7	0.9300
C3—C4	1.409 (7)	C1—C2	1.499 (7)
C8—C7	1.390 (7)		
			
O2—S1—O4	111.1 (3)	C3—C4—H4	121.2
O2—S1—O3	114.0 (3)	C7—C6—C5	122.0 (5)
O4—S1—O3	113.1 (3)	C7—C6—H6	119.0
O2—S1—O1	103.0 (4)	C5—C6—H6	119.0
O4—S1—O1	108.5 (3)	S1—O1—H1	104 (7)
O3—S1—O1	106.3 (2)	C4—C5—C6	121.9 (5)
C2—N1—C3	108.6 (4)	C4—C5—H5	119.1
C2—N1—H1A	125.7	C6—C5—H5	119.1
C3—N1—H1A	125.7	C6—C7—C8	116.5 (4)
C2—N2—C8	108.5 (3)	C6—C7—H7	121.8
C2—N2—H2A	125.8	C8—C7—H7	121.8
C8—N2—H2A	125.8	F2—C1—F3	110.5 (7)
N1—C3—C8	107.2 (4)	F2—C1—F1	108.3 (6)
N1—C3—C4	132.5 (4)	F3—C1—F1	105.2 (5)
C8—C3—C4	120.3 (5)	F2—C1—C2	112.0 (5)
C7—C8—N2	132.7 (4)	F3—C1—C2	111.0 (4)
C7—C8—C3	121.9 (4)	F1—C1—C2	109.5 (5)
N2—C8—C3	105.4 (4)	N1—C2—N2	110.3 (4)
C5—C4—C3	117.5 (5)	N1—C2—C1	125.9 (4)
C5—C4—H4	121.2	N2—C2—C1	123.8 (4)

### Hydrogen-bond geometry (Å, °)

**Table:**

*D*—H···*A*	*D*—H	H···*A*	*D*···*A*	*D*—H···*A*
N2—H2A···O3^i^	0.86	1.85	2.707 (5)	173
N1—H1A···O4	0.86	1.88	2.740 (7)	174
O1—H1···O2^ii^	0.86 (2)	1.86 (6)	2.608 (7)	145 (9)

Symmetry codes: (i) *x*+1, *y*, *z*; (ii) *y*−1, −*x*+*y*, *z*+1/6.
